# Structural Characterisation of the Beta-Ketoacyl-Acyl Carrier Protein Synthases, FabF and FabH, of *Yersinia pestis*

**DOI:** 10.1038/srep14797

**Published:** 2015-10-15

**Authors:** Jeffrey D. Nanson, Zainab Himiari, Crystall M. D. Swarbrick, Jade K. Forwood

**Affiliations:** 1 School of Biomedical Sciences, Charles Sturt University, Wagga Wagga, NSW, 2678, Australia; 2 E.H. Graham Centre for Agricultural Innovation, Charles Sturt University, Wagga Wagga, NSW, 2678, Australia

## Abstract

*Yersinia pestis*, the causative agent of bubonic, pneumonic, and septicaemic plague, remains a major public health threat, with outbreaks of disease occurring in China, Madagascar, and Peru in the last five years. The existence of multidrug resistant *Y. pestis* and the potential of this bacterium as a bioterrorism agent illustrates the need for new antimicrobials. The β-ketoacyl-acyl carrier protein synthases, FabB, FabF, and FabH, catalyse the elongation of fatty acids as part of the type II fatty acid biosynthesis (FASII) system, to synthesise components of lipoproteins, phospholipids, and lipopolysaccharides essential for bacterial growth and survival. As such, these enzymes are promising targets for the development of novel therapeutic agents. We have determined the crystal structures of the *Y. pestis* β-ketoacyl-acyl carrier protein synthases FabF and FabH, and compared these with the unpublished, deposited structure of *Y. pestis* FabB. Comparison of FabB, FabF, and FabH provides insights into the substrate specificities of these enzymes, and investigation of possible interactions with known β-ketoacyl-acyl carrier protein synthase inhibitors suggests FabB, FabF and FabH may be targeted simultaneously to prevent synthesis of the fatty acids necessary for growth and survival.

The gram negative bacterium *Yersinia pestis*, causative agent of bubonic, pneumonic, and septicaemic plague, remains a major public health threat. Responsible for three human pandemics; the Justinian plague (sixth to eighth centuries), the Black Death (fourteenth to nineteenth centuries), and modern plague (nineteenth century to the present day), *Y. pestis* remains endemic in many parts of North America, South America, south east Asia, and Africa^1^, with outbreaks occurring in China^2^, Madagascar^3^, and Peru^4^ in the last five years.

As pneumonic plague, *Y. pestis* is highly contagious and able to spread from human-to-human through respiratory droplets, averting the normal zoonotic route of infection in which plague is spread through contact with infected fleas^5^,^6^. *Y. pestis* can also remain viable in soil for at least 24 days^7^, and in bottled water for 72–160 days, dependent on the strain^8^. If left untreated, *Y. pestis* infections are usually fatal, resulting in death in as little as 24 h after onset of symptoms^5^,^9^,^10^. These characteristics illustrate the potential of *Y. pestis* as a biological weapon, with the USA Centre for Disease Control and Prevention classifying this bacterium as a Category A bioterrorism threat.

In this context, reports of antimicrobial resistance in *Y. pestis* are alarming, with evidence of two multiple drug resistant (MDR) strains displaying high level resistance to ampicillin, chloramphenicol, kanamycin, streptomycin, spectinomycin, sulfonamides, tetracycline, and minocycline, agents commonly used for either treatment or prophylaxis^11^,^12^. The lethality of untreated infections, the highly contagious nature of pneumonic plague, and the potential of *Y. pestis* as a biological weapon, combined with the emergence of MDR strains, illustrates that *Y. pestis* poses a major threat to public health, and the need for new antimicrobial agents to treat drug resistant strains.

The type II fatty acid biosynthesis (FASII) system of bacteria, required by many bacteria for the synthesis of essential lipoproteins, phospholipids, and lipopolysaccharides, is an attractive target for drug discovery. In contrast to the multi-domain mammalian type I fatty acid synthase (FASI) complex, each reaction of the FASII pathway is catalysed by a discrete enzyme (see Zhang, *et al*.^13^, Cronan and Thomas^14^, and Parsons and Rock^15^ for comprehensive reviews). The β-ketoacyl-acyl carrier protein (ACP) synthases, FabB, FabF, and FabH, catalyse the Claisen condensation of fatty acyl-thioesters and malonyl-ACP to form a β-ketoacyl-ACP intermediate elongated by two carbon atoms. The initial cycle of elongation is catalysed by FabH, involving condensation of malonyl-ACP and acetyl-CoA, while subsequent cycles of elongation are performed by FabB or FabF. Interestingly, whilst FabB and FabF have overlapping substrate specificities, FabB appears necessary in *Escherichia coli* for elongation of the 10-carbon unsaturated *cis*-3-decenoyl-ACP intermediate formed by FabA, a crucial role in unsaturated fatty acid synthesis, with *E. coli* FabF unable to perform this reaction. Conversely, FabF appears necessary for thermal regulation of membrane fluidity, and the elongation of palmitoleate (C16:1) to *cis*-vaccenate (C18:1). However, FabF enzymes from bacteria that do not possess a FabB homologue are able to fulfil the role of FabB, implying an evolution of these enzymes to incorporate both activities^16^,^17^,^18^.

Despite catalysing different reactions of the FASII pathway, FabB, FabF, and FabH all share a similar active site architecture and reaction mechanism. The active site of all three enzymes consists of a catalytic triad centred around a cysteine residue, with FabB and FabF enzymes both possessing a Cys/His/His catalytic triad^19^,^20^, while FabH possesses a Cys/His/Asn catalytic triad^21^,^22^. Importantly, these structural similarities may permit the design of new antimicrobials able to target multiple β-ketoacyl-ACP synthases simultaneously, as has been observed with platencin, platensimycin, and thiolactomycin, which have been shown to inhibit the FASII condensing enzymes of *E. coli*, *Mycobacterium tuberculosis*, and *Staphylococcus aureus* with varying degrees of success^23^,^24^,^25^,^26^,^27^,^28^,^29^,^30^. Cerulenin has also been shown to inhibit FabB and FabF enzymes, but is a poor inhibitor of FabH^23^,^29^,^31^,^32^. An inhibitor targeting all three β-ketoacyl-ACP synthases simultaneously would effectively eliminate synthesis of the fatty acids necessary for growth and survival.

Here we describe the crystal structures of the *Yersinia pestis* β-ketoacyl-ACP synthases FabF and FabH, and compare these with the unpublished, deposited structure of FabB, presenting three promising targets for the development of new antimicrobials to combat MDR *Y. pestis* strains.

## Materials and Methods

### Cloning

The genes encoding *Yp*FabF (accession no. YP_002346612.1) and *Yp*FabH (accession no. YP_002346608.1) were amplified from genomic DNA by PCR using HotStarTaq PCR Master Mix (Qiagen) and cloned into the expression vector pMCSG21, encoding a N-terminal hexahistidine tag with a *Tobacco etch virus* (TEV) protease cleavage site, via ligation-independent cloning as described previously^33^,^34^.

### Expression and purification

Recombinant *Yp*FabH and *Yp*FabF were expressed as His-tagged fusion proteins in *E. coli* BL21(DE3) pLysS cells. Briefly, 5 ml Luria-Bertani (LB) broth supplemented with spectinomycin (100 μg ml^−1^) and chloramphenicol (34 μg ml^−1^) was inoculated and incubated overnight at 310 K and 220 rev min^−1^. This culture was used to inoculate auto-induction medium (Studier, 2005) containing spectinomycin (100 μg ml^−1^) and chloramphenicol (34 μg ml^−1^), which was incubated overnight at ~298.15 K and 90 rev min^−1^. Cells were harvested by centrifugation and resuspended in His buffer A (50 mM phosphate buffer pH 8.0, 300 mM NaCl, 20 mM imidazole). The His-tagged proteins were purified by affinity chromatography (HisTrap HP column, GE Healthcare), unbound proteins were removed by extensive washing with His buffer A, and recombinant protein was eluted using an increasing concentration gradient of His buffer B (50 mM phosphate buffer pH 8.0, 300 mM NaCl, 500 mM imidazole). Following elution of protein, fractions containing recombinant *Yp*FabF or *Yp*FabH were incubated with TEV protease (~0.1 mg ml^−1^) for 14 h at ~277 K to cleave the His-tag. Target proteins were further purified by size-exclusion chromatography (S-200 column, GE Healthcare) and eluted in 50 mM Tris pH 8.0, 125 mM NaCl. Fractions containing the purified protein were concentrated using an Amicon ultracentrifugal device (Millipore) with a 10 kDa molecular-weight cut-off. The concentrated proteins were assessed by SDS-PAGE to be >90% pure, and stored at 193.15 K.

### Crystallisation

Initial crystallisation screens were performed using a range of commercially available crystal screens (Crystal Screen, Crystal Screen 2, PEG/ION, and PEG/ION 2 from Hampton Research; PACT Premier and Proplex from Molecular Dimensions). Crystal screens were performed in VDX 48-well plates (Hampton Research) via the hanging drop vapour diffusion technique, using 1.5 μl recombinant *Yp*FabH or *Yp*FabF solution combined with 1.5 μl reservoir solution, suspended over 300 μl reservoir solution, and incubated at 296 K.

Feather shaped *Yp*FabH crystals were observed in Hampton Research Crystal Screen condition 41 (10% Propan-2-ol, 0.1 M HEPES sodium salt pH 7.5, 20% PEG4000). *Yp*FabF crystals were obtained in multiple conditions from the Molecular Dimensions PACT premier crystallisation screen (conditions 19, 20, 21, 32, 33, 43). To obtain single diffraction quality crystals, crystallisation conditions were optimised around different molecular weights and concentrations of PEG, and by varying salt and additive concentrations. Diffraction quality *Yp*FabH crystals were formed in 10% Propan-2-ol, 0.1 M HEPES sodium salt pH 7.5, 15% Glycerol, 24% PEG4000, using a protein concentration of 20 mg ml^−1^. Acetylated-*Yp*FabH crystals were produced by co-crystallisation with acetyl-CoA at a molar ratio of ~5 moles of acetyl-CoA and ~5 moles of malonyl-CoA to one mole of *Yp*FabH, using the above conditions and a protein concentration of 10 mg ml^−1^. Diffraction quality *Yp*FabF crystals were obtained in 0.2 M lithium chloride, 0.1 M HEPES sodium salt pH 7.0, 24% PEG6000, using a protein concentration of 21 mg ml^−1^.

### Data collection and structure determination

Prior to data collection, *Yp*FabH and *Yp*FabF crystals were cryoprotected in 20% glycerol and flash-cooled in liquid nitrogen at 100 K. Diffraction data were collected at the Australian Synchrotron. Raw data were indexed and integrated using XDS (*Yp*FabH/acetylated *Yp*FabH)^35^ or iMosflm (*Yp*FabF)^36^, and scaled in Aimless^37^ from the CCP4 suite^38^,^39^. The structures of *Yp*FabH and *Yp*FabF were solved by molecular replacement using Phaser^40^ and a monomer of *E. coli* FabH (PDB:1HN9) and *E. coli* FabF (PDB:1KAS) as search models for *Yp*FabH and *Yp*FabF respectively. Successive rounds of model building were performed in Coot^41^ and refined using Phenix^42^. A summary of the crystallographic and refinement statistics for apo-*Yp*FabH, acetylated *Yp*FabH, and *Yp*FabF are provided in Table 1.

### Docking simulations, sequence alignments, and protein interface analysis

Docking of platencin, platensimycin, and thiolactomycin to *Yp*FabH was performed using the SwissDock web service (http://www.swissdock.ch/)^43^. FabH and FabF sequence alignments were generated using the T-Coffee (http://tcoffee.crg.cat/)^44^,^45^ and ESPript (http://espript.ibcp.fr/)^46^ web services. Protein interfaces and monomer-monomer interactions were assessed using the Protein interfaces, surfaces and assemblies’ service (PISA), at the European Bioinformatics Institute (http://www.ebi.ac.uk/pdbe/prot_int/pistart.html)^47^.

## Results and Discussion

### Diffraction data and structure solution

*Yp*FabH and *Yp*FabF were cloned, recombinantly expressed as 6xHis-tagged fusion proteins in *E. coli*, purified by a two-step purification incorporating affinity and size exclusion chromatography, and crystallised. *Yp*FabH, acetylated-*Yp*FabH, and *Yp*FabF diffraction data were integrated using XDS and iMosflm, and scaled in Aimless to a resolution of 2.20 Å, 1.80 Å, and 2.70 Å respectively.

Apo*-Yp*FabH crystals displayed C222_1_ symmetry, with the unit cell parameters *a *= 91.35, *b *= 120.02, *c *= 49.30 Å, and were estimated to contain one *Yp*FabH molecule in the asymmetric unit, with a solvent content of 38.5% and a Matthews coefficient of 2.00 Å^3^ Da^−1^. Acetylated *Yp*FabH crystals displayed the same symmetry as apo-*Yp*FabH crystals, and similar unit cell dimensions (see Table 1). *Yp*FabF crystals displayed P12_1_1 symmetry, with the unit cell parameters *a *= 74.67, *b *= 63.91, *c *= 89.29 Å, α* *= 90, β* *= 107.14, c* *= 90°. Based on a molecular weight of 45,500 Da, the asymmetric unit was estimated to contain two *Yp*FabF molecules, with a solvent content of 45.09% and a Matthews coefficient of 2.24 Å^3^ Da^−1^.

The crystal structures of *Yp*FabH and *Yp*FabF were solved by molecular replacement, using a monomer of *E. coli* FabH (PDB:1HN9) and *E. coli* FabF (PDB:1KAS) as the search models respectively. The final structure of apo-*Yp*FabH was refined to an R_work_ of 17.2% and R_free_ of 22.1%, acetylated *Yp*FabH to an R_work_ of 14.8% and R_free_ of 17.9%, and *Yp*FabF to an R_work_ of 19.9% and R_free_ of 24.5%.

### Overall structure of *Yersinia pestis* FabH

**Figure 1:**
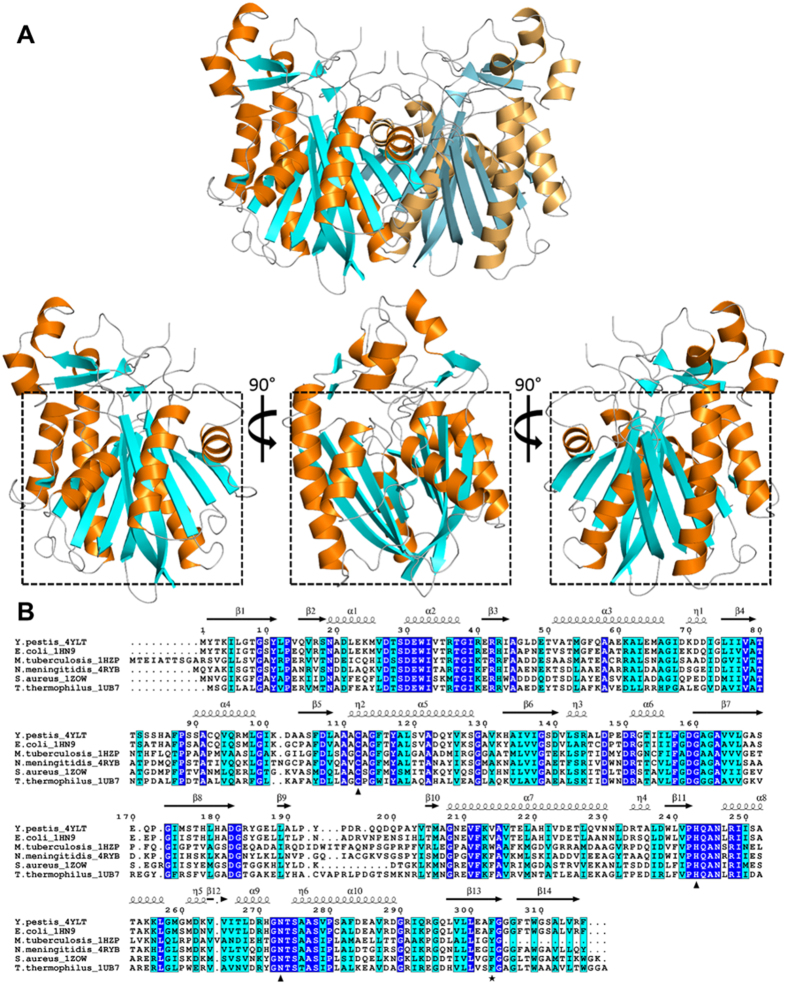
The structure of *Yersinia pestis* FabH (*Yp*FabH). **(A**) A cartoon representation of *Yp*FabH as a dimer, and a monomer at 0°, 90°, and 180° of rotation around the horizontal axis, with the thiolase fold motif highlighted by a black square. β-sheets are displayed in cyan, α-helices are displayed in orange. (**B**) Sequence alignment of *Yp*FabH with FabH from *E. coli* (PDB:1HN9), *M. tuberculosis* (PDB:1HZP), *N. meningitidis* (PDB:4QAV), *S. aureus* (PDB:2GQD), and *T. thermophilus* (PDB:1J3N). Strictly conserved residues are highlighted in blue with white text, similar residues are highlighted in cyan, residues of the active site catalytic triad are designated by triangles, residue Phe303, which is thought to play a role in substrate specificity, is designated by a black star. Secondary structure features of *Yp*FabH, including α-helices, β-sheets, and 3-10 helices (η) are shown above the sequence alignment in black.

**Figure 2:**
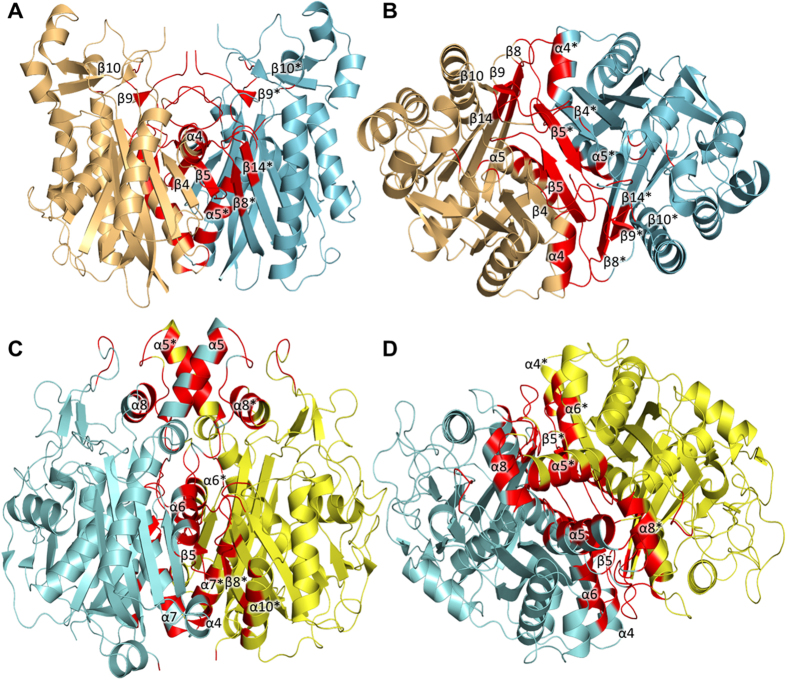
*Yersinia pestis* FabH (*Yp*FabH) and *Yersinia pestis* FabF (*Yp*FabH) dimer interfaces. Secondary structure features of the *Yp*FabH dimer interface at 0° **(A)** and 90° **(B)** of rotation around the vertical axis. The most significant interactions of the *Yp*FabH dimer interface occur between β5 and α5, which pack against their counterparts of the opposing monomer forming a 10 stranded β-sheet that spans to two monomers, and α4, which packs against β8, β9, β10, and the adjacent loop regions of the opposing monomer. The *Yp*FabF dimer interface at 0° **(C)** and 90° **(D)** of rotation around the vertical axis. The most prominent interactions of the *Yp*FabF dimer interface occur between strand β5, helix α7, and the connecting loops (residues ~160–180) of each monomer, with helix α7 (residues ~170–180) and strand β5 (residues 157–158) interacting with this loop and their counterparts of the opposing monomer. Further interactions are formed between α7 and α10; α4 and α6 that lie against the loop region connecting β8 and α10 of the opposing monomer, and α5 which packs against helices α5 and α8 of the opposing monomer. Interface residues are highlighted in red, opposing monomer secondary structure features are indicated by asterisks (*).

The final model of *Yp*FabH contains a monomer in the asymmetric unit with 10 α-helices and 14 β-strands. The N-terminal and C-terminal halves of *Yp*FabH share a high degree of symmetry, with the two halves arranging to form a thiolase fold motif characteristic of the FASII condensing enzymes^19^,^20^,^48^. The thiolase fold consists of two five stranded mixed β-sheets flanked either side by two α-helices with the topology α-β-α-β-α, in which each α represents two α-helices, and each β represents a five stranded mixed β-sheet (Fig. 1). Based on analysis using PISA, the biological unit of *Yp*FabH is predicted to be a dimer, which is consistent with that observed in bacterial homologues. The dimeric assembly is related by the symmetry operator x,-y,-z, with the two subunits forming a dimer interface burying approximately 15.6% (~1,965 Å^2^) of the total surface area. The most extensive monomer–monomer interactions are formed between β5 of each subunit which arrange anti-parallel, creating a 10-strand β-sheet that spans the dimer, and a network of hydrogen bonds formed between β5 and α5 (residues 105–130), and the equivalent residues of the opposing monomer, and helix α4, which interacts with β8, β9, and β10 (residues 180–200) of the opposing monomer (Fig. 2A,B).

### Overall structure of *Yersinia pestis* FabF

**Figure 3:**
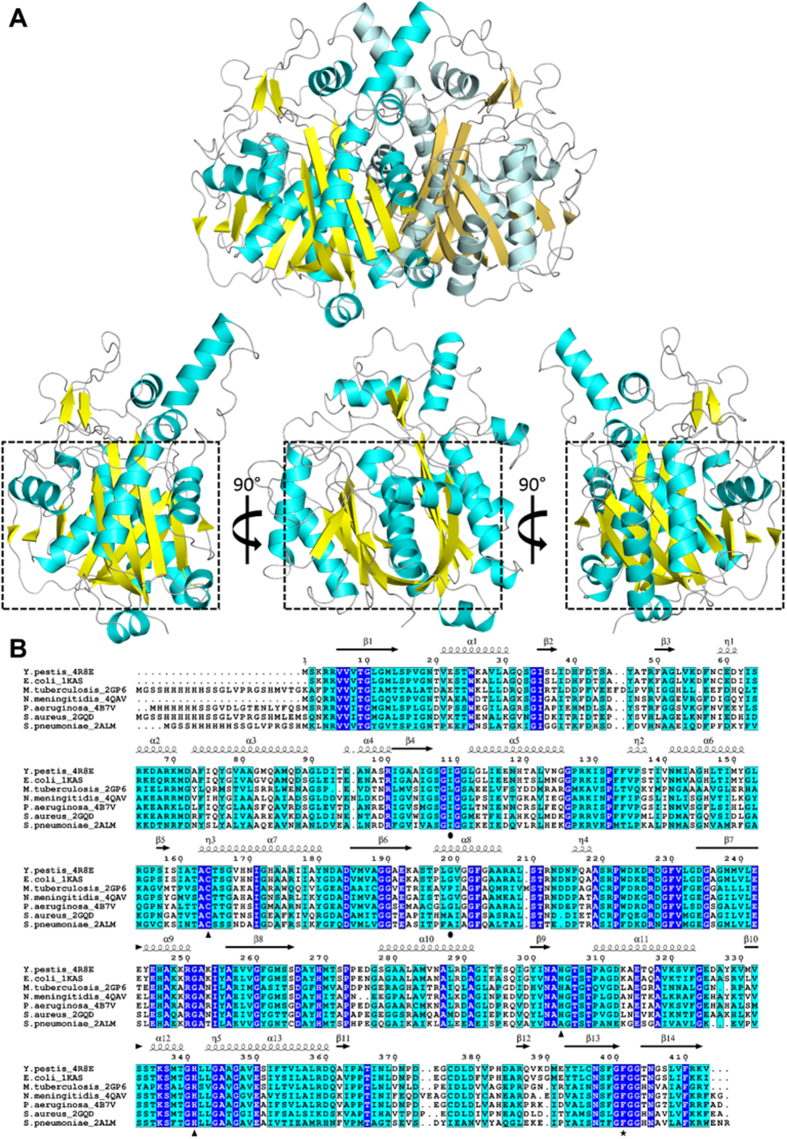
The structure of *Yersinia pestis* FabF (*Yp*FabF). (**A**) A cartoon representation of *Yp*FabF as a dimer, and a monomer at 0°, 90°, and 180° of rotation around the horizontal axis, with the thiolase fold motif highlighted by a black square. β-sheets are displayed in yellow, α-helices are displayed in cyan. (**B**) Sequence alignment of *Yp*FabF with FabF from *E. coli* (PDB:2GDW), *M. tuberculosis* (PDB:2GP6), *N. meningitidis* (PDB:4QAV), *S. pneumoniae* (PDB:2ALM), *S. aureus* (PDB:2GQD), and *T. thermophilus* (PDB:1J3N). Strictly conserved residues are highlighted in blue with white text, similar residues are highlighted in cyan, residues of the active site catalytic triad are designated by triangles, residues Ile109 and Gly199, which are thought to direct the fatty acyl substrate, are designated by black circles, the gatekeeper residue Phe401 is designated by a black star. Secondary structure features of *Yp*FabF, including α-helices, β-sheets, and 3-10 helices (η) are shown above the sequence alignment in black.

The refined structure of *Yp*FabF contains a dimer within the asymmetric unit, with the *Yp*FabF monomer containing 13 α-helices and 14 β-strands (Fig. 3). Like the *Yp*FabH and *Yp*FabB (PDB: 3OYT, Anderson *et al*., unpublished) structures, the core motif of *Yp*FabF contains a characteristic thiolase fold, which exhibits the same α-β-α-β-α topology as that observed in *Yp*FabH. Additionally, *Yp*FabF exhibits a similar pattern of dimerisation, with strand β5 of each monomer arranged in an antiparallel fashion, however these β-strands are not positioned to form a contiguous 10-strand β-sheet as observed in *Yp*FabH (Fig. 3). The *Yp*FabF monomers are related by a two-fold crystallographic axis with the most prominent interactions occurring between strand β5, helix α7, and the adjoining loop regions (residues ~160–170) of each monomer, with helix α7 (residues ~170–180) and strand β5 (residues 157–158) interacting with this loop and their counterparts on the opposing monomer. Additional interactions are formed between α7 and α10, α4 and α6 that pack against the loop region connecting β8 and α10, and α5, which packs against helices α5 and α8 of the opposing monomer. In total, this dimer interface buries approximately 18% (~2,890 Å^2^) of the solvent accessible surface area (Fig. 2C,D).

### Active site architecture of *Yersinia pestis* FabH and FabF

**Figure 4:**
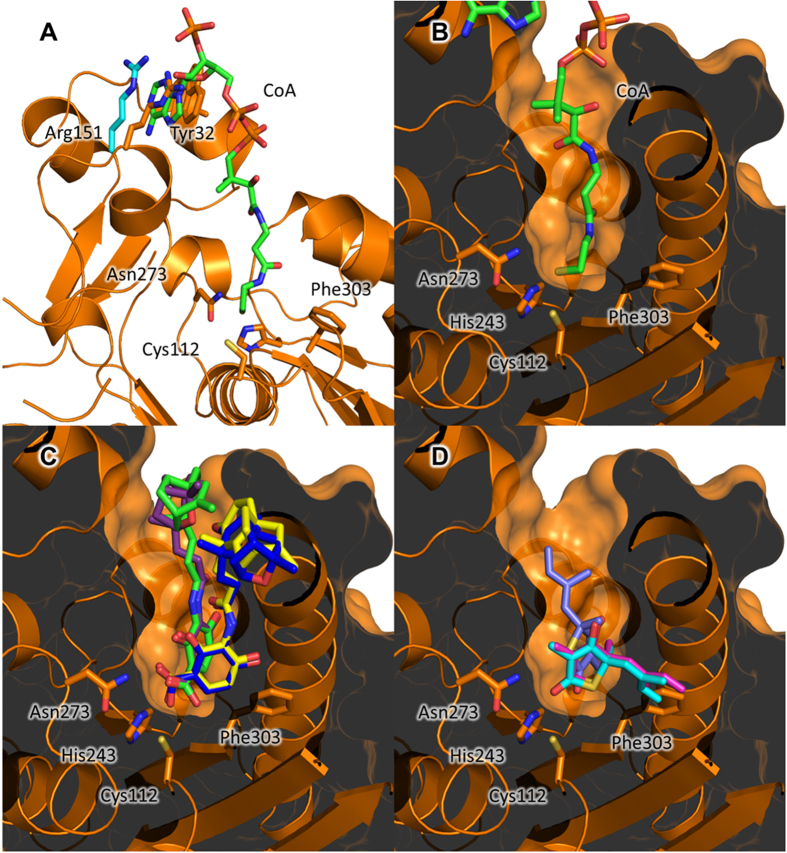
Possible substrate and inhibitor interactions of *Yersinia pestis* FabH (*Yp*FabH). **(A)** Superposition of CoA (green) from *E. coli* FabH (PDB:1HND) onto *Yp*FabH. Arg151 of *Yp*FabH clashes with the adenine ring of CoA and likely adopts a conformation similar to that observed in *E. coli* (cyan) to accommodate the substrate. **(B)** Superposition of CoA (green) also shows the phosphopantetheine arm of CoA extending the length of the binding pocket of *Yp*FabH to reach the active site cysteine. (**C**) Docking of platencin (green) and platensimycin (purple) to *Yp*FabH suggests a highly similar binding site to that observed in FabF homologues (PDB:3HO2, yellow; PDB:3HNZ, blue). **(D**) Superposition of thiolactomycin bound to FabB (PDB:1FJ4, magenta) and a *Mycobacterium* homologue (PDB:2WGE, cyan) shows the inhibitor conformation is incompatible with the *Yp*FabH binding pocket. Docking of thiolactomycin (light blue) to *Yp*FabH indicates the inhibitor rotates approximately 90°, with the cyclic motif maintaining a similar location within the active site.

**Figure 5:**
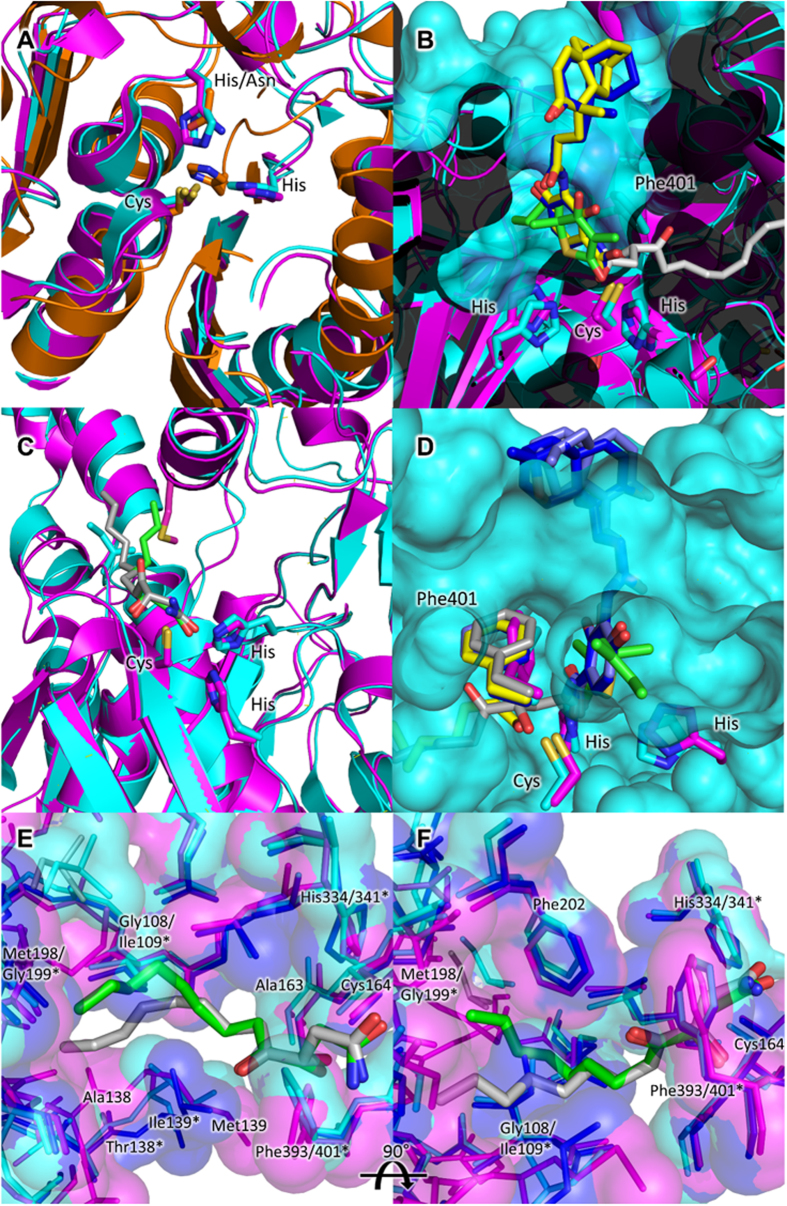
The active sites of *Yersinia pestis* β-ketoacyl-acyl carrier protein synthases and potential interactions with known inhibitors of the FASII condensing enzymes. (**A**) Superposition of the putative *Yp*FabH (orange), *Yp*FabF (cyan), and *Yp*FabB (magenta) active sites. (**B**) Superposition of cerulenin (PDB:1B3N, silver), platencin (PDB:3HO2, yellow), platensimycin (PDB:3HNZ, blue), and thiolactomycin (PDB:1FJ4, green) from bacterial FabF/FabB homologues into the active sites of *Yp*FabB (magenta) and *Yp*FabF (cyan) reveals no steric clashes or modifications which would prevent inhibition, with the exception of Phe401, which is thought to rotate into an open conformation upon substrate or inhibitor binding. (**C**) Superposition of cerulenin bound *E. coli* FabB (PDB:1FJ8, silver) and FabF (PDB:1B3N, green) showing the differing conformations of the acyl chain, and residues Ile108 of FabF (cyan) and Met198 of FabB (magenta), which direct the acyl chain of the inhibitor (cyan residue is acting upon green inhibitor, magenta residue is acting upon silver inhibitor) and possibly fatty acyl substrates. (**D**) A view from the interior of *Yp*FabF, showing access to part of the substrate binding pocket of *Yp*FabF (cyan) is closed off by Phe401. The conformation of Phe401 in FabF/FabB structures bound to cerulenin (PDB:1FJ8, silver), platencin (PDB:3HO2, light blue), platensimycin (PDB 3HNZ, blue), and thiolactomycin (PDB:1FJ4, green) closely mimic that of FabF in complex with lauroyl-CoA (PDB:2GFY, yellow). Superposition of *Yp*FabB (magenta), *Yp*FabF (cyan) and *N. meningitidis* FabF (PDB:4QAV, dark blue) active sites, and cerulenin from cerulenin bound *E. coli* FabB (PDB:1FJ8, silver) and FabF (PDB:1B3N, green) structures at 0° (**E**) and 90° (**F**) rotation around the vertical axis. Both the residues and surface of the *Yp*FabB and *Yp*FabF active sites and substrate binding pockets are highly similar. The only significant differences appear to be the replacement of Ile109 and Gly199 (Ile108 and Gly199 in *E. coli*) of FabF, with Gly108 and Met198 of FabB (magenta), and residues 138–140 of both enzymes, with no obvious differences between *Yp*FabF and FabF from *N. meningitidis*, which does not possess a FabB homologue*. Yp*FabF residues indicated by asterisks (*), *Yp*FabB and residues common to both *Yp*FabB and *Yp*FabF are not marked.

Both the active sites and reaction mechanisms of the FASII condensing enzymes have been well characterised, with FabB and FabF enzymes possessing a Cys/His/His catalytic triad, and FabH enzymes possessing a Cys/His/Asn catalytic triad. Based on structural homology with related Fab enzymes, reaction mechanisms of *Yp*FabH and *Yp*FabF are likely to follow a two-stage mechanism. Driven by a dipole moment, the active site cysteine, Cys112 and Cys164 in *Yp*FabH (Fig. 4) and *Yp*FabF (Fig. 5) respectively, attacks the acyl group of a fatty acyl donor, transferring the acyl group to the enzyme. The bound fatty acyl donor molecule is displaced, and the receiving molecule or fatty acyl thioester to be elongated binds, initiating the transfer of the acyl group from the condensing enzyme to the recipient. The remaining residues of the catalytic triad, His243 and Asn273 of *Yp*FabH (Fig. 4), and His304 and His341 of *Yp*FabF (Fig. 5), are thought to stabilise the fatty acyl intermediate during transition states^20^,^22^,^49^.

The order of substrate binding and thus reaction order in FabF and FabB is thought to be controlled by residue Phe401 (*Yp*FabF numbering) which acts as a “gatekeeper”, rotating upon acylation of the active site cysteine to expand the substrate binding pocket and allow the fatty acyl thioester, malonyl-ACP, to be elongated (Fig. 5D)^20^,^49^. While both FabB and FabF possess a similar active site phenylalanine “gatekeeper” residue, the structural elements that give rise to the differing substrate specificities of FabB and FabF are not known. Based on inspection of *E. coli* FabB and FabF structures in complex with cerulenin, Price, *et al*.^23^ suggest Gly107 and Met197 of *E. coli* FabB (Gly108 and Met198 of *Yp*FabB) direct the tail of the acyl chain toward strand β4, while the equivalent residues in *E. coli* FabF, Ile108, and Gly198 (Ile109 and Gly199 of *Yp*FabF), direct the tail of the acyl chain away from β4. As such, Gly107/Ile108 and Met198/Gly199 are thought to induce a binding pocket conformation suited to the kinked structure of the unsaturated *cis*-3-decenoyl-ACP intermediate elongated by FabB (Fig. 5C,E,F). Residues Gly107/Ile108 and Met198/Gly199 are conserved in *Yp*FabB and *Yp*FabF, however Ile108 is also conserved in the FabF enzymes of *Neisseria meningitidis, S. aureus*, and *Streptococcus pneumoniae*, which do not possess FabB homologues and rely on FabF as the sole elongating enzyme. Gly199 is also conserved in *N. meningitidis*, while this residue is replaced by alanine in *S. aureus* and *S. pneumoniae*. That Ile108 and Gly198 are conserved or replaced by a similar residue in FabF enzymes, which supplant the role of FabB, indicates that residues Gly107/Ile108 or Met198/Gly199 do not solely account for the inability of *E. coli* FabF to elongate unsaturated *cis* double bond containing intermediates produced by FabA.

The molecular basis for FabH substrate specificity has been suggested to stem from differing rotamer conformations within the substrate binding pocket. Gajiwala, *et al*.^50^ propose that different rotamer conformations, rather than amino acid substitutions or inserts observed within the binding pocket of FabH enzymes, account for differing FabH substrate specificities, with enzymes that utilise branched chain fatty acids adopting a conserved rotamer conformation equivalent to that of Phe298 in *S. aureus* FabH, Phe312 in *Enterococcus faecalis* FabH, and Tyr304 in *M. tuberculosis* FabH (Fig. 1B), where the aromatic ring of Phe/Tyr is rotated inward towards the active site, causing a reduction in the size of the hydrophobic pocket. The rotation of the equivalent residue in *Yp*FabH (Phe303) is turned away from the active site mimicking that of *E. coli*, which would suggest a similar substrate specificity to that of *E. coli* FabH. However, the relevance of the role of this rotamer conformation in *Yp*FabH substrate specificity, if any, has not yet been determined.

To further characterise the active site and substrate interactions of *Yp*FabH, we attempted to co-crystallise *Yp*FabH with acetyl-CoA. Unlike the apo-*Yp*FabH structure, the active site cysteine (Cys112) of this complex is acetylated, with no CoA phosphopantetheine moiety visible within the structure. Based on comparison with structures of *E. coli* FabH in complex with CoA (PDB:1HND, 1HNH), the absence of CoA in our acetylated-*Yp*FabH structure appears to be due to crystal packing, with crystal contacts overlapping with the 3′-ribose phosphate and ribose moiety of CoA, indicating the first stage of the reaction has taken place and the CoA molecule has been displaced from the binding pocket in anticipation of the fatty acyl recipient prior to crystallisation. Despite the acetylation of the active site cysteine, the active site appears largely unchanged, with no obvious conformational changes observed, and as the CoA phosphopantetheine moiety is absent, the interactions between this enzyme and substrate cannot be determined. However, superposition of CoA bound *E. coli* FabH structures (PDB:1HND, 1HNH) onto the structure of *Yp*FabH (Fig. 4A,B) indicates the phosphopantetheine moiety of CoA is stabilised by hydrogen bonds with Arg36, Asn209, and Asn246, while the adenine moiety appears to form a hydrogen bond with Thr28 and aromatic stacking interactions with Trp32. Superposition of these models also suggests Arg151, which clashes with the adenine moiety of CoA, moves to accommodate the substrate, with the adenine ring stacked between Trp32 and Arg151 in a planar fashion (Fig. 4A,B).

### Potential inhibitor interactions of cerulenin, platencin, platensimycin, and thiolactomycin, with *Yersinia pestis* FabB, FabF, and FabH

Despite a relatively low sequence identity (~30–40%), the overall structures of *Yp*FabB, *Yp*FabF, and *Yp*FabH are highly similar (Fig. 5A,B), with an RMSD of ~2.3 Å over ~240 residues between *Yp*FabB and *Yp*FabF, and *Yp*FabH, and an RMSD of 1.24 Å over 388 residues between *Yp*FabB and *Yp*FabF. The structural similarity of these enzymes extends to their active sites, with the inhibitors cerulenin, platencin, platensimycin, and thiolactomycin shown to inhibit multiple FASII condensing enzymes^23^,^24^,^25^. Docking of platencin, platensimycin, and thiolactomycin to apo-*Yp*FabH (Fig. 4C,D) reveals similar conformations to those observed in the crystal structures of *E. coli* FabF bound to these inhibitors (Fig. 5B), however thiolactomycin appears to rotate to avoid steric clashes with residues of the active site. Cerulenin, platencin, platensimycin, and thiolactomycin are all thought to mimic the fatty acyl thioester substrate of the FASII condensing enzymes. This is somewhat evident by the rotation of the FabF/FabB “gatekeeper” residue Phe401 in inhibitor bound complexes to a conformation equivalent to that observed in the structure of *E. coli* FabF bound to lauroyl-CoA (Fig. 5D).

**Figure 6:**
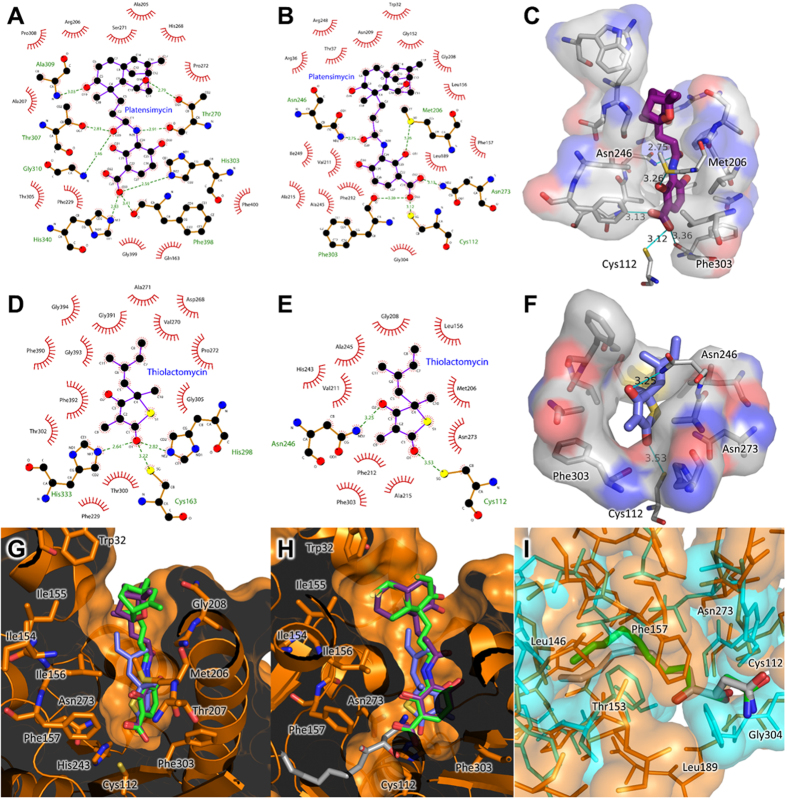
Comparison of interactions between known inhibitors of β-ketoacyl-acyl carrier protein synthases and *Yersinia pestis* FabH docked models. 2D representations of the interactions between platensimycin and *E. coli* FabF (**A**), and that of platensimycin docked to *Yp*FabH (**B**), indicate substantially fewer bonds are formed between platensimycin and *Yp*FabH compared to that of *E. coli* FabF (PDB:2GFX), and the non-polar residues Met206 and Gly208, which may repel the ether oxygen in the ketolide moiety of platensimycin. Hydrogen bonds are represented by green dashed lines, hydrophobic contacts are shown as red circular arcs. (**C**) A 3D diagram of the interactions between platensimycin and *Yp*FabH identified in Fig. 6B, showing potential hydrogen bonds (blue lines) and hydrophobic interactions (silver clouds). 2D representations of the interactions between thiolactomycin and *E. coli* FabB (PDB:1FJ4) (**D**), and that of thiolactomycin docked to *Y*pFabH (**E**), suggests the rotation of thiolactomycin to fit into the *Yp*FabH binding pocket, and the loss of dual histidine residues in the catalytic triad reduces the number of bonds which would stabilise thiolactomycin when bound to *Yp*FabH, compared to *E. coli* FabB/FabF. (**F**) A 3D diagram of the interactions between thiolactomycin and *Yp*FabH identified in Fig. 6E, showing potential hydrogen bonds (blue lines) and hydrophobic interactions (silver clouds). (**G**) A cut-away view of the *Yp*FabH (orange) active site surface showing residues that may interact with platencin (green), platensimycin (purple), and thiolactomycin (light blue). (**H**) Superposition of cerulenin (PDB:1FJ8, silver) into the active site of *Yp*FabH suggests the active site is too small to accommodate the inhibitor, which may partially account for the poor inhibition of FabH exhibited by cerulenin. (**I**) Superposition of *Yp*FabH (orange), *Yp*FabF (cyan), and cerulenin from cerulenin bound *E. coli* FabB (PDB:1FJ8, silver) and FabF (PDB:1B3N, green) structures suggests that the *Yp*FabH substrate binding pocket is shorter than that of *Yp*FabF, at least partly due to hydrophobic residues that lie near the catalytic triad, thus any future drug design efforts should accommodate for the differences in depth between the substrate binding pockets of FabB, FabF and FabH 2D representations were generated using LigPlot^+^^55^.

Whilst platencin, platensimycin, and thiolactomycin all inhibit FabH, both platensimycin and thiolactomycin inhibit FabH poorly (IC_50_ values of 67 μM and ~100 μM respectively), and cerulenin exhibits little to no inhibition of FabH. Platencin exhibits ~4 fold greater inhibitory activity (IC_50_* *= 16.2 μM) compared with platensimycin^23^,^24^,^25^,^51^, with docking of platencin and platensimycin into the active site of *Yp*FabF and *Yp*FabH showing that the terminal carboxylic acids of platencin and platensimycin form hydrogen bonds with residues His243 and Asn273 of *Yp*FabH, and His304 and His341 of *Yp*FabF. This is consistent with *E. coli* FabH docking studies performed by Jayasuriya, *et al*.^52^, who suggest the difference in inhibitory activity between platencin and platensimycin stems from interactions between FabH and the ketolide motifs of these inhibitors. The nonpolar residues Ile155, Ile156, and Trp32, posed at the entrance to the binding site of *E. coli* FabH, surround the polar ether oxygen atom of the pentacyclic ketolide motif of platensimycin. In *Yp*FabH, a similar environment is formed by residues Ile154, Ile155, Leu156, and Trp32. The ether oxygen of platensimycin in our docked models lies near the non-polar residues Met206 and Gly208 (Fig. 6), however both the conformations presented here and those proposed by Jayasuriya, *et al*.^52^ suggest unfavourable interactions. In contrast to platensimycin, the tetracyclic ketolide of platencin lacks the polar oxygen atom, allowing platencin to form favourable hydrophobic interactions with residues Trp32, Ile154, Ile155, and Leu156, or Met206, Ala207, and Gly208 (Fig. 6G,H).

The weak inhibitory activity of thiolactomycin and cerulenin against FabH, compared to that against FabF and FabB, is believed to stem from differences in the catalytic triad of these enzymes, with mutation of the Cys/His/His triad of *E. coli* FabB to a Cys/His/Asn triad similar to that observed in FabH shown to reduce the sensitivity of FabB to cerulenin by 10 fold and thiolactomycin by 14 fold^23^. Furthermore, the rotation of thiolactomycin to avoid steric clashes with the FabH active site, as evident in our docked model, may reduce the number of residues able to bind the inhibitor (Fig. 6D,F). Additionally, the substrate binding pockets of *E. coli* and *S. aureus* FabH enzymes are too short to accommodate the acyl chain of cerulenin, resulting in unfavourable steric clashes^23^,^29^ (Fig. 6H,I).

While cerulenin, platencin, platensimycin, and thiolactomycin have been shown to inhibit the FASII condensing enzymes, these molecules are not necessarily well suited for use as antimicrobials, with cerulenin, platensimycin, and thiolactomycin analogues also shown to inhibit the mammalian fatty acid synthase complex^23^,^53^,^54^. However, the use of these molecules as lead compounds for structure based drug design has the potential to yield new antimicrobials to combat MDR *Y. pestis*.

## Additional Information

**How to cite this article**: Nanson, J. D. *et al*. Structural Characterisation of the Beta-Ketoacyl-Acyl Carrier Protein Synthases, FabF and FabH, of *Yersinia pestis*. *Sci. Rep*. **5**, 14797; doi: 10.1038/srep14797 (2015).

## Figures and Tables

**Table 1:** *Yp*FabH and *Yp*FabF data and model statistics.

	apo-*Yp*FabH	acetylated-*Yp*FabH	*Yp*FabF
PDB ID	4YLT	4Z19	4R8E
Resolution range (Å)	40.80–2.20 (2.28–2.20)	49.26–1.80 (1.84–1.80)	42.55–2.70 (2.83–2.70)
Space group	C222_1_	C222_1_	P12_1_1
Unit cell length (Å)	*a *= 91.35 *b *= 120.02 *c *= 49.30	*a *= 91.46 *b *= 119.88 *c *= 49.26	*a *= 74.67 *b *= 63.91 *c *= 89.29
Unit cell angle (°)	α* *= 90° β* *= 90° c* *= 90°	α* *= 90° β* *= 90° c* *= 90°	α* *= 90° β* *= 107.14° c* *= 90°
Total observations	89126 (4038)	182940 (9341)	48943 (6683)
Unique reflections	13326 (927)	25320 (1418)	20652 (2789)
Multiplicity	6.7 (4.4)	7.2 (6.6)	2.4 (2.4)
Completeness (%)	94.5 (70.4)	99.3 (96.7)	92.6 (94.7)
Mean I/sigma (I)	17.9 (5.1)	12.2 (4.9)	4.1 (2.0)
Mean CC (1/2)	0.998 (0.943)	0.991 (0.946)	0.935 (0.457)
R-pim	0.029 (0.149)	0.046 (0.147)	0.132 (0.461)
R-meas	0.075 (0.322)	0.123 (0.383)	0.213 (0.722)
R-merge	0.070 (0.282)	0.114 (0.353)	0.166 (0.550)
R-work	0.172	0.148	0.199
R-free	0.221	0.179	0.245
Number of atoms*	2469	2564	6041
RMSD bonds (Å)	0.005	0.006	0.005
RMSD angles (°)	0.876	0.994	0.903
Ramachandran favoured (%)	97	97	96
Ramachandran allowed (%)	3	3	4
Ramachandran outliers (%)	0	0	0

^*^Note: Not including hydrogen atoms.
